# CCL13 and human diseases

**DOI:** 10.3389/fimmu.2023.1176639

**Published:** 2023-04-19

**Authors:** Laifu Li, Fei Dai, Lianli Wang, Yating Sun, Lin Mei, Yan Ran, Fangchen Ye

**Affiliations:** ^1^ Department of Gastroenterology, Second Affiliated Hospital of Xi’an Jiaotong University, Xi’an, China; ^2^ Shaanxi Province Key Laboratory of Gastrointestinal Motility Disorders, Laboratory of Digestive Diseases of the Second Affiliated Hospital of Xi'an Jiaotong University, Xi’an, China

**Keywords:** CCL13, MCP-4, cytokines, human diseases, Th2, NF-κB, type 2 immunity

## Abstract

CCL13/MCP-4 belongs to the CC chemokine family, which induces chemotaxis in many immune cells. Despite extensive research into its function in numerous disorders, a thorough analysis of CCL13 is not yet accessible. The role of CCL13 in human disorders and existing CCL13-focused therapies are outlined in this study. The function of CCL13 in rheumatic diseases, skin conditions, and cancer is comparatively well-established, and some studies also suggest that it may be involved in ocular disorders, orthopedic conditions, nasal polyps, and obesity. We also give an overview of research that found very little evidence of CCL13 in HIV, nephritis, and multiple sclerosis. Even though CCL13-mediated inflammation is frequently linked to disease pathogenesis, it’s fascinating to note that in some conditions, like primary biliary cholangitis (PBC) and suicide, it might even act as a preventative measure.

## 1 Introduction

**Figure 1:**
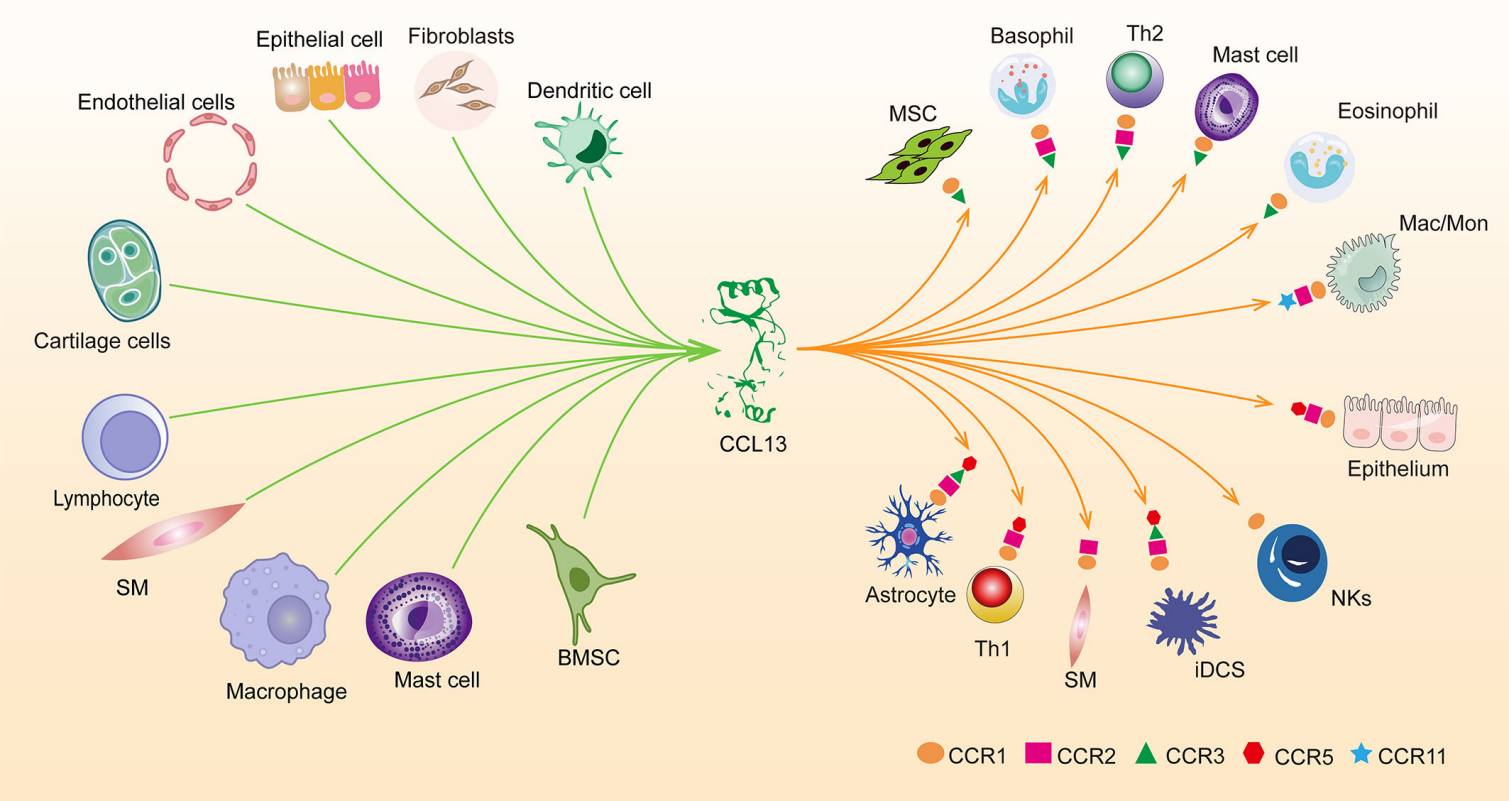
Overview of cells releasing CCL13 at the protein level and the expression of five receptors for CCL13 in cells. smooth muscle cells (SM), Macrophages (Mac), Monocytes (Mon), immature dendritic cells (iDCs), Natural killer (NKs), Helper T Lymphocyte (Th), Bone Marrow Stromal Cells (BMSC).

Chemotactic cytokines are divided into four subfamilies based on how their amino-terminal (N-terminal) cysteines are arranged: CXC, CC, XC, and CX3C; their main function is to induce directional cell migration or the migration of cells drawn to chemotactic factors towards the source of the chemotactic factor along the signal of increased chemotactic concentration. CC chemotactic factor family member CCL13, also known as MCP-4 (monocyte chemoattractant protein 4) (1). CCL13 can bind to CCR1, CCR2, CCR3, CCR5, and CCR11, causing eosinophils, monocytes, T cells, and immature dendritic cells to migrate (2) (**Figure 1**). In addition to its chemotactic activity, CCL13 has been shown to induce eosinophil degranulation, basophil histamine release, adhesion molecule expression, and secretion of pro-inflammatory cytokines in epithelial, endothelial, and muscle cells. Besides which, research has revealed that CCL13 and its derived peptides have antibacterial activity against Gram-negative bacteria (3, 4). The antimicrobial activity of cytokines may be one of the body’s defenses; therefore, elevated CCL13 in certain diseases may be associated with anti-infective properties.

Many organs, including the small intestine, thymus, colon, lung, trachea, stomach, and lymph nodes, express CCL13 at the transcription level. At the protein level, CCL13 has been reported to be present in knee chondrocytes, human proximal renal tubular epithelial cells, etc. (**Table 1**). mRNA expression of CCL13 is upregulated in various diseases, but protein-level expression has rarely been validated. Studies have demonstrated that the proteomics of CCL13 exhibits inconsistency with mRNA expression. Therefore, CCL13 mRNA-based studies require validation by protein analysis to establish convincing conclusions (5).

**Table 1:** Cells or tissues expressing CCL13 at the protein level in humans.

Locations	mRNA	Protein	Diseases	Species	References
**Turbinate tissue**	↑	-	CRSwNP	Human	(5)
**Cartilage cells**	↑	↑	Rheumatoid arthritis	Human	(6)
**Blister fluid**	not given	↑	AD	Human	(7)
**HaCaT cells**	↑	↑	AD	Human	(8)
**Peripheral blood**	not given	↑	Alopecia areata	Human	(9)
**Proximal tubular epithelial cells**	↑	↑	Glomerulonephritis	Human	(10)
**Peritubular, periglomerular**	not given	detectable	-	Human	(10)
**Corneal stromal fibroblasts**	↑	↑	Corneal injury	Human	(11)
**Nasal mucosal epithelium**	not given	↑	Rhinitis	Human	(12)
**Plasma**	not given	Late-pregnancy ↓ postnatal period ↑	Multiple sclerosis	Human	(13)
**Plasma**	not given	↑	Hodgkin lymphoma	Human	(14)
**M2 TAM**	not given	↑	OSCC	Human	(15)

AD, Atopic dermatitis; CRSwNP, Chronic rhinosinusitis with nasal polyps; and OSCC, oral squamous carcinoma; ↑: upregulation, ↓: downregulation, -: No obvious change.

**Figure 2:**
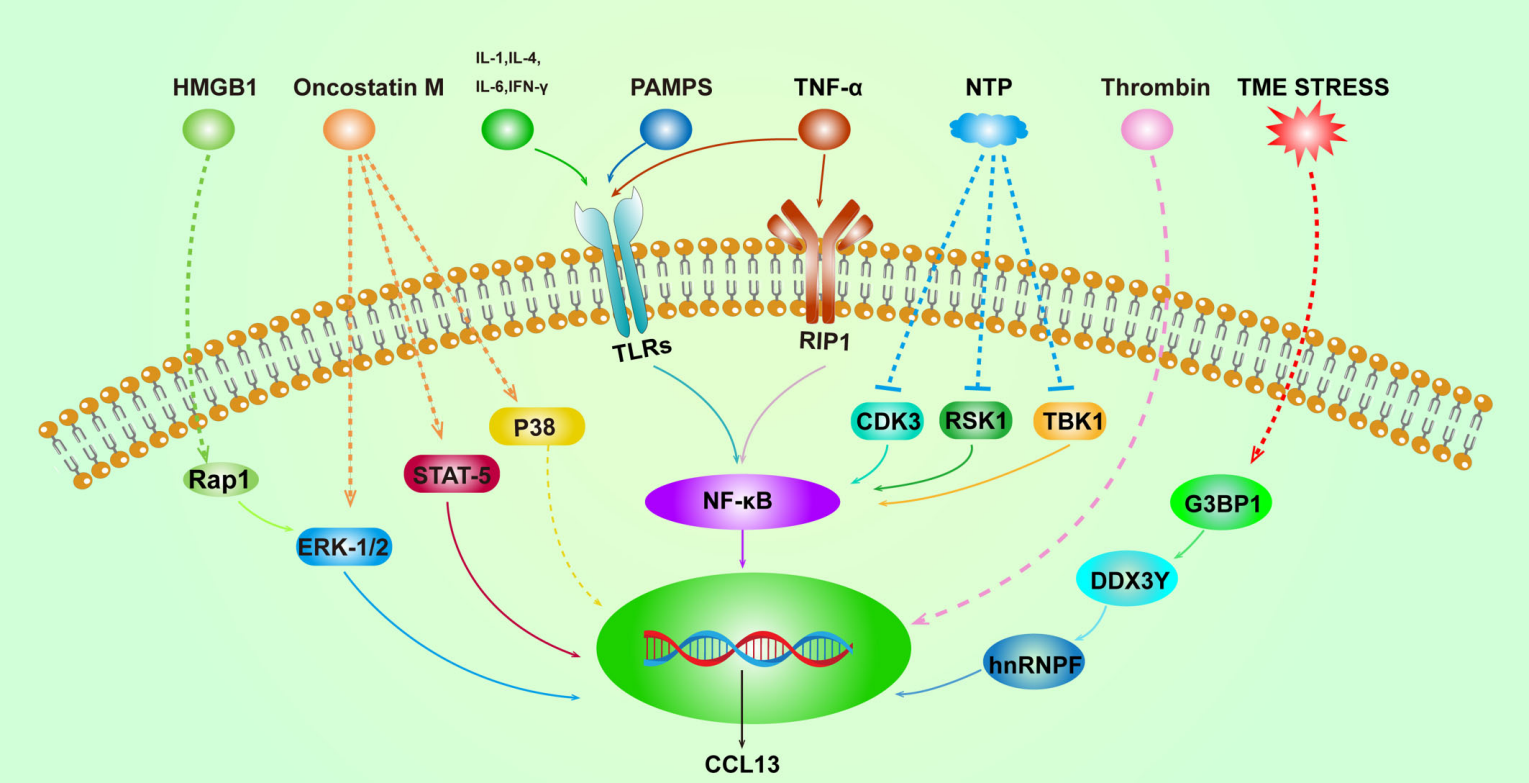
Overview of the activation pathways of CCL13. Solid lines represent well-defined relationships, and dashed lines represent intermediate molecular mechanisms unknown. High mobility group box 1 (HMGB1), Ras-Associated Protein 1 (Rap1), the tumor micro-environment (TME), and non-thermal plasma (NTP).

This article seeks to present a thorough analysis of CCL13, a summary of the function that CCL13 plays in disease, and a discussion of its probable activation pathways (**Figure 2**). Additionally, we will discuss intervention strategies that can prevent CCL13 from functioning (**Table 2**).

**Table 2:** Interventions to inhibit the function of CCL13 and their mechanisms.

Intervention means	Mechanism	Disease	Species	References
**Lebrikizumab**	IL-13 signaling was blocked by IL-13Rα1/IL-4Rα receptors	Asthma	Human	(16)
**PD98059**	Inhibition of MEK1	RA	Human	(17)
**Non-thermal plasma**	Blocking NF-κB activation in an IκB independent manner	AD	Human	(8)
**ASN002**	Inhibition of CCL13 chemotaxis through dual JAK/SYK blocking	AD	Human	(18)
**Imiquimod**	Direct inhibition of Th2-associated cytokine expression.	Hypertrophic scar	Rabbit	(19)
**Immunoglobulins**	Regulating peripheral T cell chemokines	MS	Human	(20)
**Tesamorelin**	Enhancing the GH axis to improve immune activation	AIDs	Human	(21)
**Tasquinimod**	Blocking the S100A9-TLR4 interaction deactivates NF-κB	CTCL	Human	(22)

RA, Rheumatoid arthritis; AD, Atopic dermatitis; MS, Multiple sclerosis; AIDS, Acquired Immune Deficiency Syndrome; and CTCL, Cutaneous T-cell lymphoma.

## 2 CCL13 and respiratory diseases

CCL13 and other chemokines within its family have been extensively investigated in diseases such as asthma, COPD, allergic pneumonia, and upper and lower respiratory tract infections. These diseases are characterized by inflammatory cell infiltration, which is mediated by multiple chemokines. Inflammatory cell infiltration can be triggered by pathogens or non-pathogenic factors.

### 2.1 CCL13 and asthma

Asthma is a heterogeneous lung disease with different phenotypes and unique potential mechanisms. In the past decade, people have conducted extensive research on the cellular and molecular mechanisms of asthma. The aggregation of eosinophils, type 2 helper T cells (Th2), and monocytes in the airway leads to changes in lung structure, which then leads to the decline of respiratory function. The most important pathological process of asthma is abnormal Th2 inflammation, which is mediated by Th2 cytokines, such as IL-4. About 50% of mild and moderate asthma and most severe asthma cases are induced by Th2-dependent inflammation. However, non-Th2 cytokines, including TNF-α, can also mediate Th2-low asthma (23). Airway epithelial cells can be activated by cytokines (IL-1β, IFN-γ,TNF-α, etc.) or PAMPS (Pathogen-related molecular patterns) *via* the TLRs-NF-κb pathway and releases CCL13, which recruits eosinophils and promotes the polarization of M2 macrophages to mediate the progression of asthma (2, 24, 25). When compared to patients with asthma who had their condition under control with glucocorticoids, people with uncontrolled asthma had greater CCL13 levels (26). Monocytes and eosinophils in sputum from children with asthma exhibited CCL13 and CCR3, but lymphocytes solely expressed CCL13; CCL13 is negatively correlated with peak expiratory flow and is downregulated in asthma remission (27). Elevated blood CCL13 concentrations have been observed in children with severe asthma, and it has been suggested that blood CCL13 levels can help in characterizing the severity of asthma in children (28). Mechanistically, CCL5, CCL7, CCL13, as well as CCL11 and CCL24, act synergistically to recruit eosinophils into the airways (29). Furthermore, Toll-like receptor 7 and 8 (TLR7/8) mediates the antiviral immune response by recognizing mainly viral RNA, and the increased response of the CCL13 gene to TLR7/8 agonists in the nasal mucosa of asthma patients may reflect the role of the virus in asthma progression (30). Interestingly, CDIP-2, which is a peptide derived from CCL13, has been shown to reduce chemokine-mediated function, decrease leukocyte recruitment, and reduce cytokine production by interacting with CCR1, CCR2, and CCR3 (31, 32). These results imply that CDIP-2 has the potential to alleviate airway inflammation and could be a promising therapeutic target for asthma. IL-13 is known to play a key role in the pathogenesis of asthma, and its upregulation has been associated with increased levels of CCL13 in the serum of asthma patients; Using Lebrikizumab, a monoclonal antibody that blocks the IL-13Rα1/IL-4Rα receptors, has had good results in lowering the serum concentrations of CCL17, CCL13, and total IgE in asthma patients (16). Yet it should be highlighted that, as shown in another trial, a single blockage of IL-13 was found to be insufficient to improve lung function in asthma patients who were not getting inhaled steroids (33). Bcl6 appears to be a promising inflammatory substance that inhibits histone acetylation of the chromatin of the gene cluster in lung epithelial cells by binding to a specific site of the CCL13 gene, leading to the downregulation of CCL13 (34).

The role of the CCL13-Th2 axis in promoting the entry of M2 macrophages and eosinophils into the airways and triggering airway inflammation in asthma is well established. While targeting type 2 cytokines to downregulate CCL13 has shown effectiveness, blocking upstream mediators of CCL13 alone has not been proven to be sufficient in achieving favorable outcomes.

### 2.2 CCL13 and COPD

Chronic Obstructive Pulmonary Disease (COPD) is a serious chronic respiratory condition which is currently the third greatest cause of death worldwide (35). Airway inflammation and damaging emphysema characterize COPD. In terms of pathology, it entails infiltration of inflammatory cells, modification of the airways, and irreversible damage of the lungs, among these, chronic inflammation is essential for the emergence of COPD (36). CCL13 has been linked to the development of COPD and has been shown to significantly increase when human lung tissue is stimulated by respirable smoke extracts from cooking (37). Contrarily, analysis of macrophages from bronchoalveolar lavage fluid (BALF) of COPD patients revealed primarily non-polarized macrophages with reduced gene expression of CD163, CD40, and CCL13, which are involved in pathogen recognition and processing; non polarized macrophages are primarily to blame for the decreased phagocytic capacity and the reduced ability to recognize and handle pathogens in lung macrophages (38). Moreover, a study by Ghebre et al. (39) revealed that the microbiome spectrum in sputum from patients with asthma and COPD seems to reflect different lineages of inflammatory mediators, and the upregulation of CCL13 and other type 2 mediators suggests the possibility of the infection of the bacterial phylum Bacteroidetes; type 1 mediators such as CXCL10 are more closely related to the phyla Actinobacteria and Firmicutes; pro-inflammatory mediators such as IL-1β are increased following the bacterial phylum Proteobacteria infection; these inflammatory lineages imply heterogeneity in bacterial related exacerbations of asthma and COPD, and suggest that endotype may be more important than the diagnosis of the disease itself. However, Proteobacteria, Actinobacteria, and Firmicutes are associated with other types of media. At last, bronchoalveolar lavage and biopsy samples’ content of CCL13 and other MCP family members may not be specific in identifying asthma, tuberculosis, nodular disease, and chronic bronchitis (40).

The different stages of the illness may play a role in the contradictory findings of CCL13 in the progression of COPD. CCL13 may add to chronic inflammation and tissue damage in the early phases of COPD. The detection and removal of pathogens by macrophages, as well as tissue healing and regeneration, may be promoted by CCL13 in later phases, when the lung tissue is already damaged. It’s also conceivable that CCL13’s dual function is context-dependent and affected by a variety of elements, including the pathogens involved, the host immune system, and additional environmental variables. Clarification of CCL13’s function in the development of COPD and its possible medicinal uses requires more study.

### 2.3 Acute eosinophilic pneumonia and hypersensitivity pneumonitis

Acute eosinophilic pneumonia (AEP) is a condition characterized by a significant infiltration of eosinophils in the lungs. Interestingly, eosinophil migration in endothelial cells is only induced by AEP-BALF, but not BALF from patients with hypersensitivity pneumonitis. The accumulation of eosinophils in AEP is mediated by CCR3 ligands, such as CCL13 and, CCL24 (41). The CCR3 antagonist YM-355179 has been shown to have potential in the treatment of eosinophil-associated allergic inflammatory disorders by blocking chemokine-mediated intracellular Ca^2+^ influx; this mechanism prevents the accumulation of eosinophils in affected tissues, thereby reducing the severity of the associated inflammation (42).

Although CCR1 and CCR3 are receptors for CCL13-induced eosinophil-triggered allergies, it is crucial to remember that CCR1 also has a function in CCL13-induced allergic pneumonia. Further investigation into the role of CCR1 in this mechanism may shed light on the etiology of allergy illnesses linked to eosinophils and possibly reveal novel targets for therapy.

### 2.4 Acute upper and lower respiratory tract infections

Acute lower respiratory infection (ALRI) is a prevalent infectious disease that affects individuals of all ages. ALRI in young children are known to be most frequently brought on by the respiratory syncytial virus (RSV) (43). According to a study, levels of CCL13 were found to be higher in patients with ALRI who were hospitalized from the start to day 6 after admission, but it wasn’t correlated with the severity of their symptoms; in fact, it was even downregulated in severe cases of patients with a CURB-65 score of >3 (suggestive of severe pneumonia) (44). Based on the available evidence, the relationship between CCL13 and ALRI remains unclear. While some studies suggest a potential role for CCL13 in ALRI, there is currently no definitive evidence linking the expression level of CCL13 to the severity of ALRI symptoms, further research is needed to better understand the potential role of CCL13 in the pathogenesis and clinical course of ALRI. Finally, In upper respiratory tract infections, CCL13 and CCL7 have been suggested to play a role in the recruitment of macrophages in children with confirmed viral infections of the upper respiratory tract (45).

## 3 CCL13 and rheumatism

### 3.1 CCL13 and RA

Rheumatoid arthritis (RA) is a chronic inflammatory disease that is one of the most prevalent autoimmune disorders. The disease primarily affects the joints, resulting in inflammation of the synovial membrane, which can cause joint swelling, stiffness, and pain (46). Serum CCL13 levels and expression in synovial and cartilage tissues are elevated in RA patients. The joint is the main site of inflammation in RA, and the cells known to produce CCL13 in the joint are synovial fibroblasts and chondrocytes (6, 47). Synovial fibroblasts promote chondrocyte catabolism and the development of synovial bone fragmentation, and they also have an aggressive inflammatory, stromal regulatory, and invasive character. These elements work together to cause joint destruction (48). Oncostatin M (OSM) is necessary for IL-6 and TNF- to positively regulate CCL13 expression in synovial fibroblasts. STAT-5, ERK-1/2, and p38 are involved in the production of CCL13 in response to OSM, and the increased CCL13 inhibits H_2_O_2_-induced apoptosis in synovial fibroblasts, enhances intra-synovial macrophage infiltration and angiogenesis, and aids in the course of the disease (47, 49). Studies have also demonstrated that the MAPK pathway mediates CCL13-induced proliferation of synovial fibroblasts and that these effects can be completely inhibited by PD98059 (17). In rheumatoid arthritis, estrogen has been found to decrease synovial fibroblast death and increase CCL13 expression, and this process may explain why women are more likely than males to get RA (50). In cartilage tissue, CCL13 is substantially expressed at both the mRNA and protein levels. CCL13, which is released by chondrocytes and aids in the deterioration of joints in RA, encourages the formation of rheumatoid synovial cells (6). Hintzen et al. (47, 49) reported that whereas all types of examined fibroblasts showed evidence of efficient OSM-induced signaling, only synovial fibroblasts were found to release CCL13. Although synovial fibroblasts are the primary source of CCL13 in the context of RA, several studies have demonstrated that CCL13 can be produced in response to different stimuli by other types of fibroblasts, such as dermal fibroblasts, colonic subepithelial myofibroblasts, and nasal polyp fibroblasts (51–53).

### 3.2 CCL13 and OA

Inflammation is one of the key mechanisms in the complicated joint condition known as osteoarthritis(OA), and imaging is a crucial way to diagnose OA (54). Inflammatory molecules known as the danger-associated molecular pattern (DAMP) contribute to the inflammatory process in OA joints. Alkaline calcium phosphate crystals, which are OA-associated DAMPs, can downregulate the expression of CCL13 and MRC1 (M2 markers) in macrophages (55); however, clinical studies have shown that CCL13 levels in serum and synovial fluid (SF) correlate with the severity of OA as determined by imaging (56). Patients with KL4 knee OA have significantly higher levels of CCL13 in serum and SF than those with KL2 and KL3, and patients with KL3 knee OA have significantly higher levels of CCL13 in SF than those with KL2 (The KL grading system is the most commonly used to assess the severity of joint disease in patients with OA, with grade 0 being the normal knee and grade 4 being the most severely affected) (56). It is evident that CCL13 exhibits distinct expression patterns in OA, and this pattern of expression in macrophages merits exploratory research.

### 3.3 CCL13 and other rheumatic diseases

Sjogren’s syndrome (SS) is an autoimmune disease, antibodies associated with SS can upregulate the expression of CC chemokines (CCL2, CCL13, and CCL20) in human salivary gland epithelial cells *via* the TACE/TNF-α/NF-κB signaling pathway (57). Nevertheless, in a cross-sectional study comparing several illnesses, serum CCL13 levels were measured in patients with systemic sclerosis (SSc), dermatomyositis, systemic lupus erythematosus (SLE), and healthy individuals, and it was discovered that only SSc patients had elevated levels of serum CCL13 compared to the control group (58, 59). In conclusion, while CCL13 cannot be used to diagnose rheumatism, it may be coupled with other disease-specific markers to increase the accuracy of the diagnosis.

## 4 CCL13 and skin diseases

Atopic dermatitis (AD) and alopecia areata are the two primary dermatological disorders linked to CCL13 that have been described in the literature, with the former being more clearly linked.

### 4.1 CCL13 and AD

Atopic dermatitis (AD) is a prevalent chronic inflammatory skin disease. The development of AD is influenced by various factors, including genetic predisposition, skin barrier dysfunction, and innate and adaptive immune dysregulation (60). CCL13 has been extensively studied as a Th2-associated marker in various tissues, including human PBMCs, dendritic cells, macrophages, and lesional skin tissue from patients with AD. However, the findings have not always been consistent. In lesional skin tissue from AD patients of all ages, CCL13 mRNA expression is significantly increased and has been shown to contribute to IgE synthesis (61–63). Furthermore, in older AD patients, CCL13 is considered a risk factor for atherosclerosis, indicating that these people might gain from screening for and treatment of cardiovascular disease (64). Proteomic analysis of blister fluid in AD patients has shown that CCL13 is one of the most upregulated proteins (7). However, the basal mRNA expression of CCL13 is downregulated in the PBMCs of AD patients, and CCL13 secretion into PBMCs is significantly increased upon stimulation with TLR2 ligands (65). It has been suggested that age-specific therapies may be beneficial for AD because CCL13 levels decrease significantly in diseased tissue and blood With increasing age (64). Targeting CCL13 for AD treatment is in its infancy, and an *in vitro* experiment (8) has pointed out that TNF-α promotes CCL13 gene expression in both IκB dependent and non-dependent pathways by binding to RIP1(receptor interacting protein 1); Non-thermal plasma (NTP) inhibited NF-κB pathway in a non-IκB-dependent manner and downregulated CCL13 expression in AD mice, and the combination of NTP and 1% hydrocortisone cream was found to be more effective. In 2018, the FDA authorized the oral medication ASN002, a dual JAK/SYK inhibitor, for the treatment of moderate to severe atopic dermatitis; ASN002 significantly reverses the transcriptome of lesional skin to a nonlesional phenotype and inhibits key inflammatory pathways involved in the pathogenesis of AD, including CCL13-mediated Th2-associated inflammation (18).

Indeed, age is a significant factor in determining the function of CCL13 in AD, and more investigation is required to understand why CCL13 expression in AD-lesioned tissues and PBMC differs.

### 4.2 CCL13 and alopecia areata

Alopecia areata is a common autoimmune disorder characterized by the immune-mediated destruction of hair follicles. One of the main events in the etiology of alopecia areata is the loss of hair follicle immune privilege, although the underlying processes are complicated and may involve both local and systemic immune dysregulation (66). Alopecia areata has traditionally been thought to be associated with Th1 activation, but recently there is ample evidence that Th2 may also mediate the development of alopecia areata. GWAS studies suggest a role for Th2-associated genes in the pathogenesis of alopecia areata (67). Elevated Th2 markers in the blood and skin of patients with alopecia areata may be involved in systemic inflammation and could be a potential indicator of the severity of the disease (9, 68). Upilumab, an IL-4R inhibitor, blocks the Th2 axis in patients with alopecia areata, and these patients showed downregulation of Th2-related markers (CCL13, CCL18, CCL26, CCL24) starting at week 12 of treatment with dupilumab, significant upregulation of the hair keratin gene-set at week 24, and clinical improvement in patients with alopecia areata at week 48 (69). This demonstrates how Th2-related cytokines can suppress the expression of hair keratins and be harmful in alopecia areata. To ascertain the function of particular Th2 inhibition in the treatment of alopecia areata, larger size and longer clinical trials are still necessary before the Th2 axis may be targeted for the treatment of alopecia areata.

## 5 CCL13 and digestive system disease

### 5.1 CCL13 and hepatic disease

According to Townsend et al.’s research, CCL13 expression is reduced in hepatitis D patients; this change in chemokine levels may contribute to the faster disease development in hepatitis D patients (70). On the other hand, secondary bacterial infections in cirrhotic individuals may be linked to the increase of CCL13 transcript levels in their serum and duodenal mucosa (71). Although little is known about CCL13’s role in the development of cirrhosis, recent research suggests that it may slow the advancement of the disease through type 2 immunity. However, viruses can counteract this effect in a number of ways. Elevated CCL13 may be a protective factor and needs to be further investigated, given that Gram-negative bacteria are more frequently seen in bacterial infections associated with cirrhosis.

Primary biliary cholangitis (PBC), is a chronic autoimmune liver disease characterized by cholestasis and the presence of anti-mitochondrial antibodies in the bloodstream (72). It was shown that Th2 chemokines were downregulated in early PBC, where serum CCL13 was elevated in early and decreased in late of PBC, and negatively correlated with PBC staging (r = -0.373), preventing early disease progression (73). Given the marked eosinophil infiltration in the portal vein in patients with early PBC, the increased CCL13 in the serum of patients with early PBC might be associated with this, and the downregulation of CCL13 in the late stages seems to indicate that it is not involved in the subsequent progression of PBC.

### 5.2 CCL13 and IBD

Inflammatory bowel disease (IBD) is an idiopathic inflammatory disease of the intestinal tract that primarily includes ulcerative colitis (UC) and Crohn’s disease (CD). Although its pathogenesis is unclear, chronic lesions of CD show an excessive Th1 response and a Th2 pattern is present in the mucosa of lesions in early CD and UC (74). Studies have shown that the effects of pharmacological interventions alone on chemokines in patients with IBD appear to exhibit targeting. Only CXCL10 was significantly downregulated in the blood of patients with CD treated with atorvastatin, while the other eight chemokines, including CCL13, were not significantly changed (75). Interestingly, in a clinical study that included UC and CD, after treatment with vedolizumab, although serum CCL13 levels increased in the treatment group as a whole; when patients were subdivided into groups with and without responders, CCL28 was down-regulated in responders, whereas CCL13 was up-regulated in non-responders, and they concluded that CCL13 levels at the initiation of treatment may predict the potential prognostic value of vedolizumab (76). The mechanism of CCL13 in IBD may be related to its induction of adhesion molecule expression in epithelial cells, and increased expression of adhesion molecules allows more cells of the innate immune system (such as monocytes or neutrophils) to enter the site of inflammation. The greater CCL13 levels in non-responders following therapy may be partially explained by this.

## 6 CCL13 and renal diseases

Studies on individuals who experienced acute renal allograft rejection and vasculitis glomerulonephritis revealed that CCL13 was primarily expressed in peritubular, periglomerular, and perivascular sites and was linked to the infiltration of CD3^+^ lymphocytes and CD68^+^ monocytes/macrophages. Proximal tubular epithelial cells also displayed low levels of CCL13 protein expression, which could be seen by protein blotting. The upregulation of CCL13 expression is a result of pro-inflammatory cytokines in response and plays an important role in monocyte/macrophage recruitment and retention in renal inflammation (10). In addition, kidney donor quality significantly affects renal transplantation outcomes, such as recipients of kidney donors from the elderly who exhibit low post-transplant renal function and short graft lifespan; a single-center, retrospective, observational study showed that compared to standard criteria donors and living donors, elderly and expanded criteria donors have upregulated CCL13 at the transcriptional level and may be involved in post-transplant renal inflammation and renal function impairment (77).

## 7 CCL13 and ocular disorders

Ocular uveitis can be a symptom of a variety of connective tissue disorders, including seronegative spondyloarthritis (SpA), nodular disease, pseudoarthrosis, recurrent polychondritis, and granulomatous polyangiitis. Yet uveitis that manifests as SpA has its own distinctive features, notably with a strong link to the presence of HLA-B27 (78). For this reason, uveitis presenting with spondyloarthritis is collectively referred to as B27-associated uveitis, which typically develops in young adults but can also affect children and adolescents. Studies have shown that people with HLA-B27-associated uveitis have 255-fold higher levels of CCL13 in their aqueous humor than healthy individuals. Additionally, compared to granulomatous uveitis, non-granulomatous uveitis exhibits a considerably higher level of CCL13 (79). This suggests that CCL13 is involved in the pathogenesis of non-granulomatous uveitis and that the associated immune responses may be more effective in this type of uveitis, particularly in HLA-B27-associated uveitis.

Patients with ischemic retinal vein occlusion (RVO) had modestly elevated levels of vitreous CCL13, which correlated with vitreous hemorrhage and may have indicated how severe the retinal inflammation was (80). In patients with primary rheumatogenic retinal detachment (RD), CCL13 levels are higher in vitreous fluid (VF) than in macular holes (MH), and several cytokines seem to be involved in the immune initiation and profibrotic processes after RD, and these cytokines may be related to the increased intraocular fluid volume and higher mobility of the fluid around the refractive apparatus of the anterior chamber (81). Furthermore, *in vitro* experiments have demonstrated that treatment of corneal stromal fibroblasts with thrombin resulted in a significant increase in CCL13 mRNA expression (up 2-fold) and protein production (from 0 to 14 pg/ml) and that CCL13 played a crucial role in mediating the thrombin-triggered immune response (11).

## 8 CCL13 and nasal polyps and rhinitis

Nasal polyposis (NP) is an inflammatory disease with Th2 skewing, and it is widely believed that certain chemotactic agents and Th2 factors play a crucial role in its pathogenesis. One study showed that fibroblasts may be a major source of Th2 chemokines and that TLR2, 3, 4, and 5 ligands can synergistically induce the production of CCL13 in nasal polyp fibroblasts when combined with IL-4, while TLR7/8 or 9 ligands do not induce its production (53). The expression of Th2 markers, including CCL13, was not found to be altered in nasal polyposis (NP) patients receiving glucocorticoid (GC) therapy; this lack of change in expression levels may suggest that T-cell-driven NP inflammatory mediators are resistant to the effects of GC treatment (82).

Chronic rhinosinusitis with nasal polyps (CRSwNP) is a condition characterized by a type 2 immune response, with overproduction of IgE and infiltration of eosinophils (83). Much of the research on the immunopathology of CRSwNP has relied on transcriptomics. However, Workman et al. (5) noted that proteomic analysis can reveal inconsistencies with mRNA expression, with tissue proteomic and transcriptomic analyses of CRSwNP polyp tissue found significantly elevated CCL13 mRNA levels, but no change in protein expression, and this implies that research based on mRNA of CCL13 should be supported by protein.

Allergic rhinitis is also an inflammatory disease characterized by excessive production of local type 2 cytokines and increased eosinophils in tissues. In patients with seasonal allergic rhinitis, under nasal allergen stimulation, the level of CCL13 in nasal secretions increases 3.7-fold, while IL-10 and IL-4 significantly decrease; CCL13 may worsen, while IL-10 may alleviate nasal mucosa allergy (12). In addition, similar to asthma, increased responsiveness of CCL13 to TLR7/8 agonists has also been observed in allergic rhinitis. The response to nasal administration of Resiquimod (a specific TLR7/8 agonist) can be used to simply assess nasal mucosal responsiveness (30).

## 9 CCL13 and obesity and its complications

In recent years, much interest has been generated in the fundamental mechanisms causing the global rise in obesity. Many health issues related to obesity, including coronary atherosclerosis, have been linked in studies to the emergence of persistent low-grade inflammation in the body (84). A positive energy balance and an increased anabolic state, especially in adipocytes, may be the initial stimulus in the case of obesity. This results in the release of chemokines that activate an adaptive inflammatory response, which encourages the healthy expansion of adipocytes while lowering energy stores (85).

It has been demonstrated that adipocytes from obese individuals exhibit higher expression of CCL13 compared to those from lean individuals (86), and elevated levels of CCL13 are positively associated with BMI (87, 88). According to *in vitro* studies, CCL13 expression in preadipocytes is low at baseline but steadily rises following differentiation (89).

Integrated analysis of miRNA and genome-wide data from epicardial adipose tissue (EAT) in patients with coronary artery disease (CAD) suggests that altered metabolic and inflammatory regulation is a hallmark of EAT in CAD. Furthermore, miR-103-3p/CCL13 appears to be a novel candidate that plays a role in EAT function and CAD (90). Additionally, CCL13/CCR2 has been shown to promote the formation of carotid plaques and may act as a link between the activation of platelets and monocytes (91). Coincidentally, data from mouse models confirm that when fed a high-fat diet, mice lacking CCR2 eat less and are less likely to become obese, and CCR2 antagonist therapy lowers inflammatory aspects of obesity, such as macrophage infiltration in adipose tissue (92). Earlier on, a study of adipokines in obesity and related comorbidities suggests that CCL13, a brand-new biomarker for extreme obesity, may exacerbate subclinical atherosclerosis in persons with obesity by affecting circulating levels of major atherosclerotic markers. After bariatric surgery, CCL13 was significantly reduced, indicating that reduced levels of CCL13 may be one of the mechanisms leading to cardiovascular risk reduction in obese patients following bariatric surgery (93).

Together with CAD, obesity has become a significant risk factor for periodontitis. Notably, levels of CCL13 and high-sensitivity C-reactive protein (hs-CRP) were found to be significantly elevated in both gingival crevicular fluid and serum, and a positive correlation between CCL13 and periodontal parameters (94). When the relationship between obesity, chronic periodontitis, and serum CCL13 concentrations was examined, Pradeep et al. (95) discovered that obese patients with chronic periodontitis had significantly higher serum CCL13 concentrations than the non-obese group. All these discoveries implies that CCL13 and hs-CRP may be markers of chronic inflammation in obesity and periodontal disease.

## 10 CCL13 and tissue repair

Wound healing is a complex process that occurs in response to skin damage, and involves a series of reparative events that aim to restore the protective skin barrier (96). When the wound is inflamed, the probability of forming a hypertrophic scar will greatly increase (97, 98). CCL13 is barely expressed in normal scar formation but is expressed for a longer period in hyperplastic scar formation (99). An animal study showed that Imiquimod significantly inhibited the expression of CCL13 mRNA and upregulated Th1-associated chemokines in rabbits at 21 days to 63 days after surgery to reduce collagen deposition and the extent of fibrosis and thus inhibit scar hyperplasia in a manner that regulated the Th1 and Th2 axes (19).

The process of bone healing is largely affected by the stability of the fixation (biomechanics) as well as the blood supply to the healing site. This repair process is composed of several phases, including inflammation, repair, and remodeling (100). High mobility group box 1 (HMGB1), a ubiquitous inflammatory factor in fractures, promotes the secretion of CCL4 and CCL13 from mesenchymal stem cells (MSCs) by activating the Ras-associated protein-1 (Rap1) signaling pathway, and these chemokines mediate the migration of MSCs, which in turn promotes fracture healing (13, 101, 102).

## 11 CCL13 and neurological disorders

Multiple sclerosis (MS) is an immune-related central nervous system disease (103). In MS, upregulation of CCL13 levels in the brain tissue and cerebrospinal fluid can induce monocyte chemotaxis and the secretion of inflammatory cytokines in lymphocytes, ultimately, causing oligodendrocyte activation and myelin destruction (20, 104). The downregulation of CCL13 protein expression in the plasma of MS patients in the third trimester of pregnancy and its increase after delivery may be related to the immune tolerance established during pregnancy, this expression pattern of CCL13 may reflect the disease activity of MS during pregnancy (14). Indeed, the haplotype gene in CCL13 is associated with susceptibility to MS in both rat and human genomes (104). In addition, the administration of intravenous immunoglobulin to patients with MS downregulates CCL13 expression in peripheral T cells, which in turn may inhibit T cell proliferation (20, 104).

## 12 CCL13 and mental illness

Mental illness refers to a range of clinical manifestations arising from brain dysfunction that is influenced by various biological, psychological, and social environmental factors, resulting in varying degrees of impairment in cognitive, emotional, volitional, and behavioral mental activities (105). In recent years, despite significant advances in molecular mechanisms in neuroscience, few biomarkers have made their way into clinical psychiatric practice (106). CCL13 has been shown to drive chemotaxis of pro-inflammatory cells to the inflamed or injured central nervous system (CNS), therefore, chemokines could potentially serve as novel diagnostic and therapeutic targets in psychiatric disorders (107).

In individuals with post-traumatic stress disorder (PTSD), there is an increase in serum levels of CCL13, CCL20, and CXCL6, which may indicate a higher risk for developing PTSD. In contrast, CX3CL1 may serve as a marker for recovery; more than any of these, CCL13 shows a positive correlation with scores on the PTSD Checklist (PCL), suggesting that CCL13 levels may be associated with the severity of PTSD symptoms (108). Another study revealed that plasma levels of CCL13/CCL2 ratios were approximately twofold higher in individuals with PTSD, without significant changes observed in cerebrospinal fluid levels, these ratios remained constant over circadian time, regardless of gender, body mass index, or age at the time of trauma, and may be potential circadian biomarkers for chronic PTSD (109). All of these suggest that the diagnosis of PTSD based on a single marker is difficult, and a combination of multiple substances may be more promising. However, unlike in PTSD, brain tissue levels of CCL13 were significantly lower in the dorsolateral prefrontal cortex of individuals who completed suicide, revealing that chemokine alterations may be suicide-specific immunological mechanisms (110). Interestingly, in a 12-year follow-up study, individuals who attempted suicide had lower levels of CCL13, CCL11, CCL4, CCL2, and CCL17 in their cerebrospinal fluid and plasma compared to those with psychosis who had never experienced suicidal thoughts (111). This long-term study strongly demonstrates that chemokines such as CCL13 are of great research value, as they are likely to play a role in the maintenance of immune homeostasis in the organism, which is critical for mood stabilization and regulation.

## 13 CCL13 and AIDS

The pathogenesis of Acquired Immune Deficiency Syndrome(AIDS) is primarily a result of cellular immunodeficiency caused by the direct and indirect actions of HIV (112). The primary targets of HIV have activated CD4^+^ T lymphocytes (113); and in untreated HIV-infected patients, the macrophage phenotype has been reported to shift from M1 in the early stages of infection to M2 in the later stages and can inhibit viral replication (114).

According to a multicenter AIDS cohort study, CCL13 detectability was found to be greater than 80%, and elevated plasma levels of CCL13 may be associated with more rapid disease progression in HIV-infected individuals (115, 116). In a multicenter AIDS cohort study (MACS) conducted from 1984 to 2009, CCL13 was found to be higher in the group of SUP (exposed to HARRT with HIV RNA suppressed to less than 50 copies/ml plasma) compared to the HAART-naive (NAI) group, on the timeline, CCL13 increased significantly in the first year of viral suppression, followed by a uniformly flat trajectory, HIV suppression appeared to increase the levels of the M2-associated chemokines (117). In addition, the growth hormone-releasing hormone (GHRH) analog tesamorelin significantly reduced CCL13 expression in HIV populations with metabolic dysregulation, and systemic and end-organ inflammation, suggesting that enhancement of the GH axis may improve immune activation in this population (21).

## 14 CCL13 and cancer

Cancer growth and response to therapy are both significantly influenced by inflammation, with chronic inflammation increasing tumor progression and therapeutic resistance. Acute inflammatory responses, on the other hand, frequently promote dendritic cell (DC) maturation and antigen presentation, which can drive anti-tumor immune responses. Multiple chemokines, such as those in the CCL and CXCL families, have been identified as key regulators of inflammation initiation and regression (118). The majority of tumor-associated macrophages (TAMs) in most solid tumors are M2-type TAMs (M2 TAMs), which are essential for controlling the immunosuppressive tumor microenvironment, encouraging tumor angiogenesis, and promoting tumor spread (15). CCL13 is a crucial chemokine for M2 TAMs and may be involved in its function of it (**Table 3**).

**Table 3:** Effect of CCL13 on phenotype in tumors or its clinical significance.

Cancer species	Significance of elevated CCL13	References
**Oral squamous cell carcinoma**	Promotion of tumor cell metastasis	(15)
**Colorectal cancer**	Markers of distant metastasis	(119)
**Gastric cancer**	Suggests tumor invasion into the submucosa	(120)
**Salivary adenoid cystic carcinoma**	Means a lower risk of recurrence	(121)
**Multiple myeloma**	Associated with β2-MG production	(122)
**Prostate adenocarcinoma**	Poor prognosis	(123)
**Breast cancer**	Promotes cancer cell proliferation	(124)
**Hepatocellular carcinoma**	Unclear	(125)
**Pediatric Hodgkin’s lymphoma**	Associated with slower response to early treatment	(126, 127)
**Cutaneous T-cell lymphoma**	Mediating immunosuppression	(22)

High blood CCL13 levels were independently a marker for predicting distant metastasis in colorectal cancer, according to logistic regression analysis, and they were substantially linked with advanced age, advanced T-stage, distant metastasis, and UICC stage in colorectal cancer (119). In cases of gastric cancer, the levels of CCL13 reflect the distinct response patterns of fibroblasts in specific tumor sites toward cancer cell invasion. Elevated levels of CCL13 can be indicative of submucosal invasion by tumor cells (120). In M2 TAMs, stress granule (SG) formation was stimulated by tumor micro-environment (TME) stress, and SG increased DDX3Y/hnRNPF mediated mRNA stability of CCL13, which in turn enhanced CCL13 expression and promoted the metastasis of oral squamous cell carcinoma metastasis, however, the above molecular expression and phenotype are reversed upon knockdown of the G3BP1 (15). Intriguingly, tissues from individuals with metastatic salivary adenoid cystic carcinoma(SACC) showed higher expression of CCL13 in the absence of tumor recurrence or perineural invasion than in the presence of tumor recurrence (121). Moreover, CCL13 is linked to 2-microglobulin (2-MG) levels in multiple myeloma, poor prognosis in prostate adenocarcinoma, and cell proliferation in breast cancer (122–124). Likewise, hepatocellular carcinoma (HCC) tumor tissues contain considerably more CCL13 mRNA than normal tissue, however this is unrelated to the clinical prognosis (125). In ovarian cancer, CCL13 triggers epithelial-mesenchymal transition *via* the p38 MAPK pathway (128). Chronic hypoxia does not seem to affect the expression of CCL13, according to *in vitro* experiments using breast cancer, hepatocellular carcinoma, and lung adenocarcinoma cells (129).

Further to that, it has been discovered that CCL13 protein expression is augmented in the plasma of pediatric Hodgkin’s lymphoma patients and is related to a sluggish early response; and for Hodgkin’s lymphoma patients, biologically-based risk stratification algorithms that incorporate CCL13 could be taken into consideration to enhance treatment results and reduce toxicity (126, 127). In cutaneous T-cell lymphoma (CTCL), CCL13^+^ monocytes/macrophages play a role in mediating immunosuppression by interacting with malignant T cells, and blocking the S100A9-TLR4 interaction with tasquinimod has been shown to inactivate the NF-κB pathway, leading to inhibition of CTCL tumor cell growth and induction of apoptosis (22).

## 15 Discussion

In summary, the main pathogenic mechanism of inflammation involves the recruitment and activation of immune cells, specifically Th2 and M2 macrophages, to inflamed tissues through the chemokine CCL13. Despite the potential of blocking the chemokine system as an important area of anti-inflammatory drug development, only a few drugs, such as ASN002 (a dual-action inhibitor of JAK/SYK), lebrikizumab (IL-13Rα1/IL-4Rα receptor blockade), maraviroc (a CCR5 antagonist), and plerixafor (a CXCR4 antagonist), have received FDA approval (16, 18, 33, 130, 131). However, a single blockade of CCL13 upstream or downstream does not appear to be effective due to high structural overlap and functional crossover between chemokines (33). Additionally, many other immune agents that can modulate chemokines are still not well studied and the molecular mechanisms are still unclear. For another, it is worth noting that CCL13 has been reported to have a positive effect on human diseases such as PBC and suicide, and further exploration of its mechanism is warranted. It should be noted that elevated CCL13 may be linked to antimicrobial resistance, and it ought to take this into account given the potential of CO infections like COPD and cirrhosis (39, 71).

It is crucial to keep in mind that while examining the connection between CCL13 and illness, age is a component that cannot be disregarded. Much research on CCL13 on both youngsters and the elderly has yielded conflicting findings (61, 64). Likewise, as some research suggests that the two may exhibit divergent expression patterns, simultaneous monitoring of lesion locations and peripheral blood chemokines is required (37, 38, 65). Furthermore, insufficient research has been done on the locations where the CCL13 protein is expressed. Several research (5, 132)indicate that CCL13 is not consistently expressed at the levels of transcription and translation, and rather than focusing exclusively on identifying changes in expression at the transcriptional level, future research should validate results through protein analysis.

## Author contributions

LL wrote the main manuscript text and prepared the figures and tables. LW made suggestions on the framework of the review. YS reviewed and revised the first draft. LM gave instructions on the drawing. YR and FY helped search literature. FD designed the study, revised and gave final approval of the manuscript. All authors contributed to the article and approved the submitted version.
